# Red and golden tomato administration improves fat diet-induced hepatic steatosis in rats by modulating HNF4α, Lepr, and GK expression

**DOI:** 10.3389/fnut.2023.1221013

**Published:** 2023-09-01

**Authors:** Rosaria Maria Pipitone, Rossella Zito, Giuditta Gambino, Gabriele Di Maria, Ayesha Javed, Giulia Lupo, Giuseppe Giglia, Pierangelo Sardo, Giuseppe Ferraro, Francesca Rappa, Daniela Carlisi, Danila Di Majo, Stefania Grimaudo

**Affiliations:** ^1^Department of Health Promotion, Mother and Child Care, Internal Medicine, and Medical Specialties (PROMISE), University of Palermo, Palermo, Italy; ^2^Department of Biomedicine, Neurosciences and Advanced Diagnostics (BIND), University of Palermo, Palermo, Italy; ^3^Euro Mediterranean Institute of Science and Technology- I.E.ME.S.T., Palermo, Italy; ^4^Postgraduate School of Nutrition and Food Science, University of Palermo, Palermo, Italy

**Keywords:** nonalcoholic fatty liver disease, golden tomato, red tomato, LEPR, NFH4a, GK, lipid homeostasis, steatosis

## Abstract

**Introduction:**

Nonalcoholic fatty liver disease (NAFLD), characterized by lipid accumulation within hepatocytes exceeding 5% of liver weight, is strongly related to metabolic disorders, obesity, and diabetes and represents a health emergency worldwide. There is no standard therapy available for NAFLD. Lifestyle intervention, including phytonutrient intake, is key in preventing NAFLD development and progression.

**Methods:**

We used a rat model of NAFLD to evaluate the effect of dietary supplementation with red tomato (RT) and golden tomato (GT)—a patented mix of fruit with varying degrees of ripeness and particularly rich in naringenin and chlorogenic acid—after steatosis development. We assessed the effects on body weight, metabolic profile, and hepatic steatosis.

**Results and discussion:**

We found a correlation between the amelioration of all the parameters and the liver gene expression. Our results showed that, together with the reversion of steatosis, the consumption of RT and GT can cause a significant reduction in triglycerides, low-density lipoprotein-cholesterol, fasting glucose, and homeostasis model assessment index. Meanwhile, we observed an increase in high-density lipoprotein-cholesterol according to the amelioration of the general lipidic profile. Regarding hepatic gene expression, we found the upregulation of Gk and *Hnf4*α involved in metabolic homeostasis, *Lepr* involved in adipokine signaling, and *Il6* and *Tnf* involved in inflammatory response. Taken together, our results suggest that dietary intake of red and golden tomatoes, as a nutraceutical approach, has potential in preventing and therapeutics of NAFLD.

## 1. Introduction

Nonalcoholic fatty liver disease (NAFLD), associated with obesity and metabolic syndrome (MetS) features, can be defined as metabolic dysfunction-associated fatty liver disease (MAFLD) (1). Currently, NAFLD is the most common chronic liver disease, with a global prevalence of ~25%. By 2030, it is expected to be the most frequent cause of liver transplantation (2, 3). NAFLD and MAFLD include a wide spectrum of clinical features from simple steatosis, which depending on comorbidities (i.e., obesity, insulin resistance, type 2 diabetes mellitus), genetic predisposition (i.e., *Pnpla3* and *Tm6sf2*), and diet and behavior (i.e., alcohol, cholesterol, fructose) can progress to nonalcoholic steatohepatitis (NASH). The latter is characterized by necroinflammation and liver fibrosis development which is associated with high-risk complications, such as liver decompensation and hepatocellular carcinoma (4, 5). The NAFLD pathogenesis and progression are complex. The recent “multiple hits” model suggests that several factors, such as lipid overload, oxidative stress, mitochondrial and endoplasmic reticulum damage, chronic inflammatory response, and hepatocyte cell death, act synergistically to enhance the development and the progression of NAFLD (6). Even though the recent progress in understanding gene profile and pathways involved in NAFLD has suggested the possible role of targeted therapies (i.e., FXR agonists and PPAR agonists), there is no approved therapy for NAFLD (7). The current treatment strategy is focused on lifestyle interventions, including increased physical activity and diet-induced weight loss. In this context, several natural compounds have attracted interest in treating NAFLD, and most attention has been given to natural products derived from fruits, vegetables, and medicinal plants (8).

Tomato (*Solanum lycopersicum* L.) is part of the Mediterranean diet, enriched in phytonutrients, mainly β- carotene, and lycopene. It also contains essential amino acids, fiber, minerals, vitamins, and monounsaturated fatty acids (9).

Today, the phytonutrient-rich Mediterranean diet is recognized for its role in preventing inflammation-based diseases such as obesity, diabetes, metabolic syndrome, and some neurodegenerative diseases (10). *In vivo* studies have shown that consuming tomatoes and their processed products protects against certain types of cancer, cardiovascular disorders, cognitive functions, and osteoporosis. Tomato phytonutrients can reduce reactive oxygen species through radical scavengers, inhibit cell proliferation and damage, modulate enzyme activity and cytokine expression, and influence signal transduction pathways (11). They also have soluble and insoluble fiber, cellulose, hemicellulose, and pectin, which can modify the intestinal microbiota and promote their fermentation. The production of short-chain fatty acids improves lipid and glycemic profiles by improving intestinal dysfunction (12). These characteristics attribute tomatoes to a protective role against cancer, diabetes, cardiovascular diseases (CVDs), and obesity (13).

The golden tomato is a product of industrial invention and is named golden peeled tomato. It is obtained by mixing fruits that are not yet fully ripe with different degrees of coloring. These products are usually considered field waste and discarded during the ripe tomato harvest. Interestingly, GT and RT have different degrees of maturation and differ significantly in terms of phytonutrients, as described in section 4.1. While GT is characterized by a higher content of naringenin, a 4,5,7-trihydroxyflavanone (57%), and chlorogenic acid (ChA)—an ester of caffeic acid and quinic acid (81%), RT has a lower quantity and quality of carotenoids as well as vitamin C (45%). Lycopene, β- carotene, naringenin, and chlorogenic acid have been shown to have hepatoprotective effects, and several activities of these phytochemicals were described to normalize BMI, improve glucose tolerance, ameliorate IR, decrease plasma and liver lipid levels, and reduce NAFLD (14–16).

The present study aims to assess the GT and RT dietary treatment effect on rats with liver steatosis to individuate possible mechanistic insight into their biological activity. We also evaluated the influence of GT and RT administration on metabolic dysfunction and hepatic steatosis induced by high-fat diet (HFD) to explore the hepatic expression of genes implicated in fatty liver disease. We evaluated the mRNA levels of genes involved in adipokine signaling and cholesterol metabolism (such as *Lepr*), carbohydrate and lipid metabolism (such as *Gk*), and inflammatory responses (such as *Il6* and *Tnf*). We evaluated the lipid homeostasis pathway with particular attention to *Hnf4*α, given this nuclear receptor's crucial role in controlling hepatic fatty deposition.

This study could shed new light on the pathways modulated by these phytonutrients at a hepatic level in NAFLD and provide knowledge for future prevention and therapeutic approaches.

## 2. Materials and methods

### 2.1. Preparation and treatment of golden and red tomatoes

**Figure 1:**
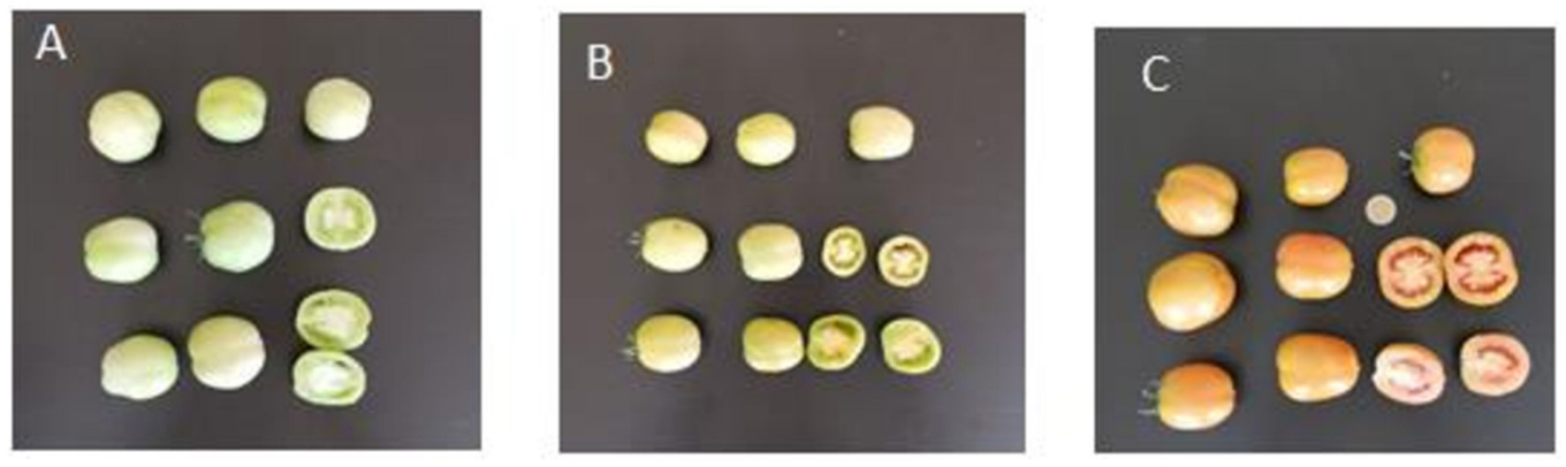
Schematic representation of different color classes of tomatoes: green **(A)**, pre-veraison **(B)**, and veraison **(C)**.

The GT sample was prepared by hybridizing fruits in different degrees of ripeness in appropriate proportions. It is a patented product registered as “golden peeled tomato” with the number 102015000058418. The ripening degree of the fruits corresponding to a certain color grade was chosen using colorimetric analysis using CR-400 (Minolta Corporation, Ltd., Osaka, Japan). The fruits were collected at three different ripening stages with three different colors: green (Figure 1A), pre veraison (Figure 1B), and veraison (Figure 1C). The red tomato sample, on the other hand, was a fully ripened, red-colored fruit. All fruits come from the same cultivar Brigade, grown on the same farm, and therefore subjected to the same soil and climate conditions.

Previous work (17) has shown that GT and RT possess different qualitative and quantitative phytonutrient content and antioxidant properties. GT was found to be richer in naringenin and chlorogenic acid, while RT was richer in carotenoids and lycopene. The interest in GT products arises from using this fruit as a new functional food with different properties compared to the fully ripe product that would otherwise be discarded.

After harvesting, the GT and RT samples were divided into aliquots (~1 kg), freeze-dried, and vacuum-preserved at −20°C before being used for treating the animals.

#### 2.1.1. Tomato solutions for oral administration

The treated groups of rats received a daily dose of tomato in an aqueous solution of 200 mg/Kg body weight, which equates to a daily portion of 300 g of fresh tomato that a 70 kg man can consume. The dose was established based on valid toxicity tests for red tomatoes available in the literature (18, 19). Each animal received daily 1 ml of GT or RT (experimental groups HFD/GT and HFD/RT) solution obtained by solubilizing 50 mg of freeze-dried tomato in 50 ml of water by oral gavage through a syringe. The groups not receiving the tomato solutions took the same volume (1 ml) of plain water. No animals showed signs of toxicity or intolerance during the treatments.

### 2.2. Animals and experimental groups

**Figure 2:**
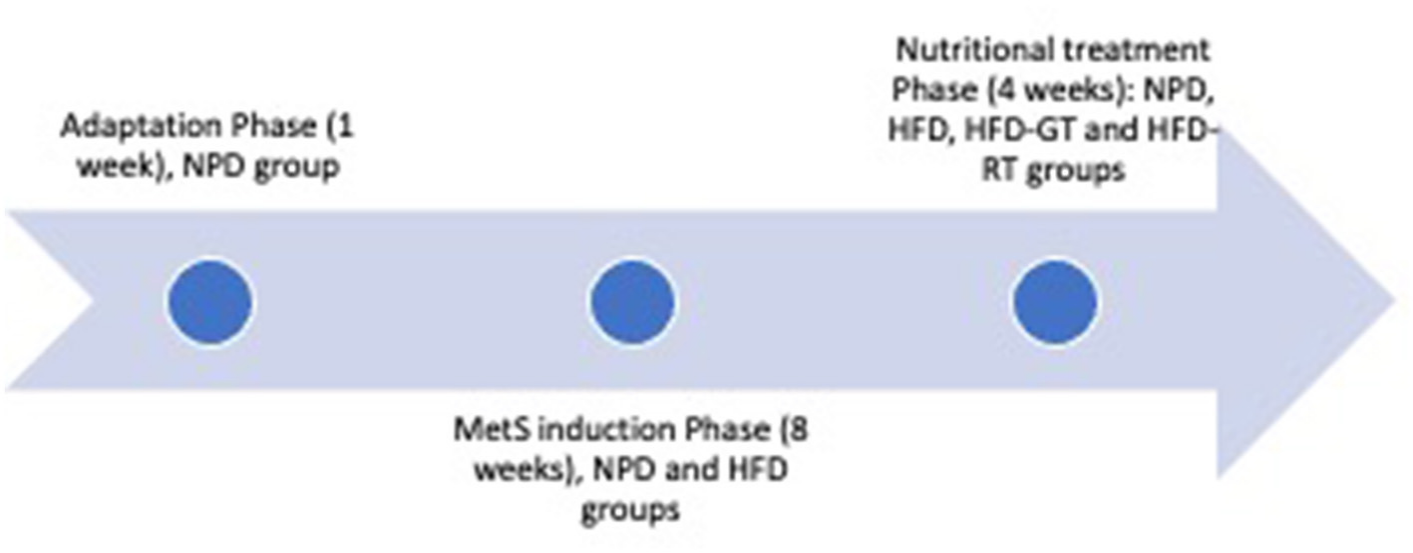
Experimental phases of the study, including adaptation phase (1 week), MetS induction phase (8 weeks), and nutritional treatment (4 weeks). The experimental groups were NPD (consuming normal pellet diet and treated with vehicle), HFD (consuming high-fat diet and treated with vehicle), HFD-GT (consuming high-fat diet and treated with golden tomato), and HFD-RT (consuming high-fat diet and treated with red tomato).

The experiments were conducted on 20 male Wistar rats (4 weeks old), with an average initial weight of 240–260 g, purchased from Envigo S.r.l. Their housing conditions in the animal facility were described in detail in our previous paper (20). During the adaptation phase of 1 week, animals were first fed with a standard chow diet providing 3.94 kcal/g and then divided into four homogenous groups with a balanced weight. The normal control group, defined as normal pellet diet (NPD, *n* = 4), was fed with a normal diet for the entire duration of the experiment, i.e., 13 weeks. The other three groups were fed high-fat diet (HFD) throughout the experiment. As indicated in Figure 2, the MetS induction phase lasted 8 weeks, at the end of which MetS was evidenced in the HFD groups according to previously identified criteria. This confirmed that the experimental groups were not different before nutritional treatment (20). The animals were treated with tomato or vehicle in the last 4 weeks of the experiment (nutritional treatment phase). Two groups representing the controls (HFD group, *n* = 5 and NPD group, *n* = 4) were orally treated with the vehicle. The other two groups were treated with golden tomato (HFD/GT group, *n* = 5) and red tomato (HFD/RT group, *n* = 5) to evaluate the effect of the golden tomato and red tomato. The experimental phases of the study are depicted in Figure 2.

### 2.3. Diet composition

The composition of both types of diet, NPD and HFD, is shown in Table 1. The animal groups were fed with standard laboratory food (code PF1609, certificate EN 4RF25, Mucedola, Milan, Italy) or with HFD food which had 60% energy coming from fats (code PF4215-PELLET, Mucedola, Milan, Italy). Fatty acid composition plays a crucial role in the regulation of metabolic phenotype. Saturated fatty acids (SFA) are more obesogenic and lead to larger significant metabolic alterations than unsaturated fatty acids. The lipid component of the HFD pellet comes from 9% palm oil consisting of 50% saturated fat, 39% monounsaturated fat, and 11% polyunsaturated fatty acids (PUFA), while the remaining fat comes from lard. The HFD is rich in saturated fatty acids in which the ratio of saturated/monounsaturated to polyunsaturated fatty acids is 4:3:1. Normally, the lipid quality consists of SFA/MUFA/PUFA in the proportions 1:1:3. All rats had free access to food. As reported, HFD induces MetS with a hepatic profile typical of NAFLD in rats after 8 weeks (21). Animal care and handling throughout the experimental procedures were in accordance with the European Directive (2010/63/EU). The experimental protocols were approved by the animal welfare committee of the University of Palermo and authorized by the Ministry of Health (Rome, Italy; Authorization Number 14/2022-PR).

**Table 1:** Components of standard laboratory food (code PF1609) and high-fat food (code PF4215).

**Component of diet**	**Pellet NPD (PF1609)**	**Pellet HFD (PF4215)**
Energy (Kcal/Kg)	3,947	5,500–6,000
Fat total (g/100 g)	3.5	34
SFA (g)	0.7	17
MUFA (g)	0.8	13
PUFA (g)	2	4
Crude protein (g/100 g)	22	23
Carbohydrates (starch g/100 g)	35.18	38
Fiber (g/100 g)	4.5	5
Ash (g/100 g)	7.5	5.5
Vitamin A (IU)	19.5	8.4
Vitamin D3 (IU)	1,260	2,100

The quantity of SFA, MUFA, and PUFA is related to the fat total of NPD or HFD.

### 2.4. Body weight gain

The influence of nutritional treatments on biometric parameters was evaluated by considering the increase in weight (body weight gain) during the last 5 weeks of the experiment. Animals were weighed once a week from the induction of MetS until the end of the nutritional treatment phase to compare the effect of GT and RT consumption on HFD-fed rats.

### 2.5. Glucose and lipid homeostasis assays

To investigate glucose tolerance, insulin resistance, and lipid homeostasis, we administered the glucose tolerance test (GTT), HOMA index, and evaluation of plasma lipid parameters at the end of the experimental protocol (21). In particular, the HOMA index was calculated by collecting plasma and serum samples for glucose and insulin according to Gambino et al. (17). Rat insulin assay determinations were done using the spectrophotometric method with kits purchased from Millipore Corporation of Merk, Germany. Blood glucose was determined by the glucose oxidase method, using glucose strips (Glucotest, Pic). HOMA index was at last determined by the formula:


$$\begin{array}{l} {\text{HOMA index }=\text{ blood glucose }\left(\text{mg}/\text{dl}\right)}^{*}\left(\text{serum insulin uU}/\text{L}\right)/405. \end{array}$$

Detailed procedures related to plasma lipid parameters have been described in our previous paper (20). In the plasma samples, triglycerides (TG), total cholesterol (TC), low-density lipoprotein cholesterol (LDL), and high-density lipoprotein cholesterol (HDL) concentrations were quantified by commercial kits using the free carpe diem device (FREE^®^ Carpe Diem; Diacron International, Italy). The data are expressed in mg/dl.

### 2.6. Determination of hepatic steatosis

Liver tissue sections (5 μm) were obtained from cryostat and stained with hematoxylin and eosin. Following staining, the slides were observed with an optical microscope (Microscope Axioscope 5/7 KMAT, Carl Zeiss, Oberkochen, Germany) connected to a digital camera (Microscopy Camera Axiocam 208 color, Carl Zeiss). The morphological evaluation of the liver steatosis was performed by a medical doctor specialized in anatomic pathology (FR), using a semiquantitative scoring system identified for NAFLD (22). This score was also used for inflammation and fibrosis that we did not observe. According to this score, steatosis was graduated from 0 to 3 where 0: 0–5%, 1: 3%−33%, 2: 34%−66%, and 3: >66%. All the observations were performed in high-power fields (HPF; magnification 400 ×), and the arithmetic means of percentage was used for statistical analyses.

### 2.7. RNA isolation and real-time PCR microarray

Total RNA was purified from the liver tissue of 15 Wistar rats using miRNeasy Micro Kit (Qiagen, Hilden, Germany) and quantified using NanoDrop™ 1000 Spectrophotometer (Thermo Fisher Scientific, Life Technologies Italia). Approximately 1 μg of RNA was retro-transcribed using the RT^2^First Strand kit (Qiagen) according to the manufacturer's instructions. Quantitative real-time PCR was performed using the pre-designed RT2 profiler PCR array rat fatty liver (96-well format, Cat. No. 330231 PARN-157ZA, Qiagen, Hilden, Germany). The plates contained primers for 84 target genes (reported in Supplementary Table 1) and for five housekeeping genes: actin beta (ACTB), beta-2-microglobulin (B2M), hypoxanthine phosphoribosyl transferase 1 (HPRT1), lactate dehydrogenase A (LDHA), and ribosomal protein, large, P1 (RPLP1). In addition, each plate contained one rat genomic DNA contamination control (RGDC), three reverse transcription control (RTC), and three positive PCR control (PPC). Data were expressed as fold regulation using the 2^−ΔΔCt^ method referred to Wistar rats fed HFD as the control group. The cycle threshold (ct) values were submitted to the Web-based PCR array data analysis software [https://geneglobe.qiagen.com/it/analyze/(Qiagen)].

### 2.8. Statistical analysis

Values of biometric, metabolic parameters, and histological evaluations of the liver were compared by a one-way ANOVA test followed by Bonferroni *post hoc* evaluations for differences between means and represented by scattered bar graphs (GraphPad Prism 9.02; San Diego, CA, USA). The body weight gains were analyzed via a two-way repeated measures (RM) ANOVA, followed by the Bonferroni *post hoc* test for significant differences in intra-subject comparisons, considering the effect of “time” and “treatment” and their interaction in the experimental groups. Differences were considered significant when *p* < 0.05. The statistical power (g-power) was considered only if >0.75 and the effect size if >0.40. The results were presented as the mean ± standard error of the mean (SEM).

For determining hepatic steatosis, statistical analysis was carried out using the GraphPad Prism 4.0 package (GraphPad Inc., San Diego, CA, USA). Comparisons of histological evaluations were made using ANOVA. All data were presented as the mean ± SD, and the level of statistical significance was set at *p* ≤ 0.05.

The PCR microarray experiments and determinations were performed in each group with three Wistar rats as biological triplicates. The data were represented as mean ± SD. The statistical significance of the differences between a single group and relative control was evaluated by a two-tailed Student's *t*-test and adjusted *p*-values by Hommel's method (*p*-value cut off of 0.05 for statistical significance), and then the fold-regulation was considered a measure of biological significance. This statistical analysis was performed using R Statistical Software version 4.0.4 (23).

## 3. Results

### 3.1. Effects of red tomato and golden tomato diet on body weight

**Figure 3:**
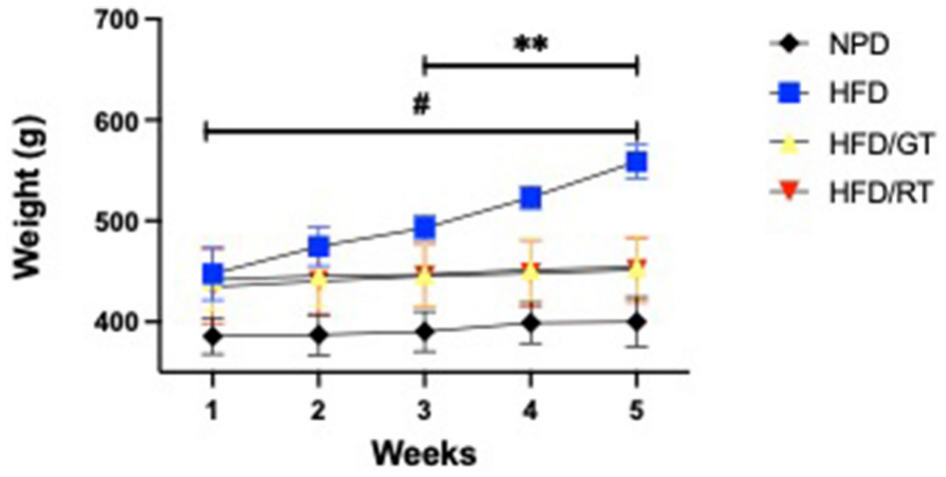
Body weight gains (g) were evaluated for 5 weeks from the MetS induction phase (week 1) to the end of the nutritional treatment phase (weeks 2–5). Statistical significance by a two-way RM ANOVA was indicated for (**) *p* < 0.001 of HFD/GT and HFD/RT vs. HFD; for (^#^) *p* < 0.001 of HFD/GT, HFD/RT, and HFD vs NPD.

The evaluation of body weight gain revealed that GT and RT treatments in rats fed with HFD reduced the biometric increase compared to rats fed on HFD alone. Significant differences in body weight were found in the experimental groups for time [*F*_(4, 60)_ = 77.62, *p* < 0.0001], treatment [*F*_(3, 15)_ = 12.66, *p* = 0.0002], and their interaction [*F*_(12, 60)_ = 30.45, *p* < 0.0001]. The *post hoc* test revealed a marked reduction of body weight gain in HFD/GT and HFD/RT vs. HFD in the fourth and fifth weeks, as indicated in Figure 3.

### 3.2. Effects of red tomato and golden tomato diet on metabolic profile

The oral supplementation of GT and RT in HFD rats was able to modify the metabolic profile in MetS.

The lipid homeostasis profile of rats is presented in Table 1. An analysis of triglycerides (TG) showed a significant reduction of TG levels in HFD/GT and HFD/RT vs. HFD [*F*_(3, 16)_: 10.86, *p* = 0.0004, g-power: 0.97, effect size: 1.32] that restored to basal values of NPD. It could appear counterintuitive that total cholesterol (T Chol) levels were much higher in HFD/GT vs. HFD, and HFD/RT was not different from HFD alone [*F*_(3, 16)_: 13.98, *p* < 0.0001, g-power: 0.95, effect size: 1.55]. However, one-way ANOVA on high-density lipoprotein cholesterol (HDL-Chol) revealed a marked increase in HFD/GT and HFD/RT vs. HFD and NPD groups [*F*_(3, 16)_: 52.77, *p* < 0.0001, g-power: 0.99, effect size: 3.49]. Further, an analysis of low-density lipoprotein cholesterol (LDL- Chol) showed a significant reduction in HFD/GT and HFD/RT vs. HFD [*F*_(3, 16)_: 8.32, *p* = 0.0015, g-power: 0.98, effect size: 1.16], like NPD basal values. Therefore, the increase in T Chol is justified by a concurrent increase in HDL and reduced LDL (Table 2).

**Table 2:** Biochemical parameters of lipid homeostasis: triglycerides (TG), total cholesterol (T Chol), LDL cholesterol (LDL Chol), and HDL cholesterol (HDL Chol) expressed as mg/dl.

**Experimental groups**	**TG**	**T Chol**	**LDL Chol**	**HDL Chol**
NPD	80.42 ± 16.08	75.44 ± 5.96	31.74 ± 9.72	26.37 ± 0.58
HFD	125.72 ± 12.83^e^	93.55 ± 4.49^d^	52.56 ± 6.00^d^	16.12 ± 2.62^d^
HFD/GT	99.17 ± 7.31^a^	110.49 ± 12.47^a, d^	34.32 ± 7.72^b^	56.56 ± 9.14^c, e^
HFD/RT	85.70 ± 16.67^b^	98.58 ± 9.60^d^	37.76 ± 4.13^a^	39.20 ± 4.95^b, d^

Statistical significance for ^a^p < 0.05, ^b^p < 0.001, and ^c^p < 0.0001 vs. HFD and for ^d^p < 0.05 and ^e^p < 0.01 vs. NPD groups.

Furthermore, glucose homeostasis was evaluated considering: (i) area under the curve (AUC), following the glucose tolerance test (GTT), and (ii) fasting glucose (FG) levels. Statistical analysis on AUC by one-way ANOVA revealed a significant main effect only for HFD/GT vs. HFD and NPD groups [*F*_(3, 16)_: 23.56, *p* < 0.0001, g-power: 0.99, effect size: 2.01; Table 2]. The plasma levels of FG were markedly reduced in HFD/GT compared to HFD [*F*_(3, 16)_: 5.74, *p* = 0.0073, g-power: 0.96, effect size: 1.09; Table 3].

**Table 3:** Biochemical parameters of glucose homeostasis, area under the curve (AUC), and fasting glucose.

**Experimental groups**	**AUC**	**Fasting glucose (mg/dl)**
NPD	333.75 ± 38.65	103.34 ± 11.17
HFD	492.79 ± 17.30^d^	150.85 ± 35.41^c^
HFD/GT	412.00 ± 18.76^b, d^	108.53 ± 11.67^a^
HFD/RT	448.40 ± 41.24	126.91 ± 9.60

Statistical significance for ^a^p < 0.05, ^b^p < 0.001 vs. HFD and for ^c^p < 0.05 and ^d^p < 0.01 vs. NPD groups.

**Figure 4:**
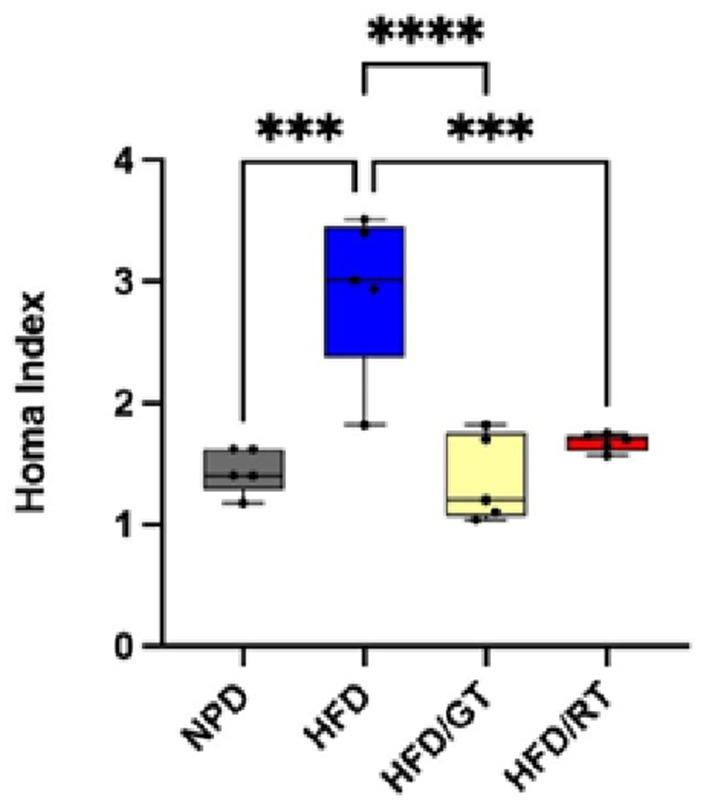
HOMA Index of the experimental groups. One-way ANOVA comparisons between groups for ***p = 0.0001 and ****p < 0.0001.

The homeostasis model assessment (HOMA) index calculation (measurement of fasting insulin and glucose) showed that the dietary supplementation with both GT and RT was able to ameliorate the insulin resistance developed after the HFD diet up to almost normal values (Figure 4). Indeed, the HOMA index revealed a marked decrease in HFD/GT and HFD/RT compared to HFD [*F*_(3, 16)_ = 17.17, *p* < 0.0001, g-power: 0.99, effect size: 1.97], returning to basal levels of NPD groups (Figure 4).

### 3.3. Effects of red tomato and golden tomato diet on hepatic steatosis

**Figure 5:**
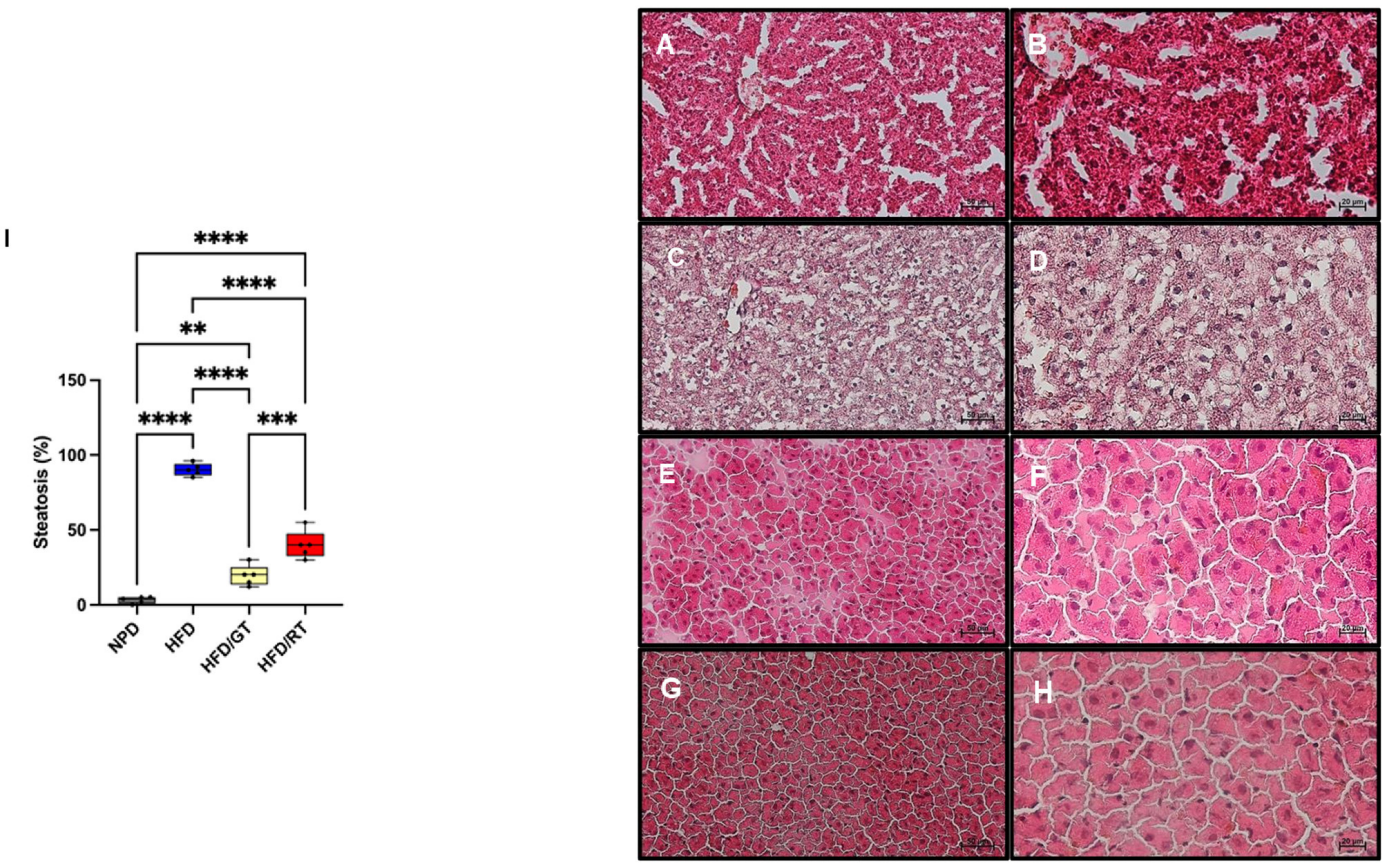
Representative images of the histological evaluation of the liver parenchyma on hematoxylin and eosin staining. [**(A, B)** Control NPD; **(C, D)** HFD; **(E, F)** HFD/RT; **(G, H)** HFD/GT]. **(A, C, E, G)** Magnification 200×, scale bar 50 μm. **(B, D, F, H)** Magnification 400×, scale bar 20 μm. **(I)** Percentage of steatosis. Data are presented as the mean ± SD. NPD vs HFD: *p* < 0.001, NPD vs HFD/RT: *p* < 0.001; NPD vs HFD/GT: *p* < 0.001; HFD vs HFD/RT: *p* < 0.001; HFD vs HFD/GT: *p* < 0.001; HFD/RT vs HFD/GT: *p* < 0.01.

The histological evaluation of liver samples from the NPD control group showed no steatosis (average percentage of 3.2 ± 0.8; Figures 5A, B) compared to the HFD group in which steatosis was found to be high (average percentage of 89.3 ± 1.5; Figures 5C, D). In HFD liver tissues, macro vesicular steatosis with diffused and large lipid droplets was predominantly observed. Liver samples of the HFD/RT group showed micro vesicular and macro vesicular steatosis, with small and large lipid droplets accumulation, in an average percentage of 43.33 ± 11.6 (Figures 5E, F). In the liver samples from the HFD/GT group, the steatosis was reduced and of micro vesicular type with a mean percentage of 20.33 ± 6.5 (Figures 5G, H). The histogram (Figure 5I) shows the percentage of steatosis in the different groups.

### 3.4. Effects of intake of red and golden tomatoes on metabolic, adipokine, and inflammatory signaling

**Figure 6:**
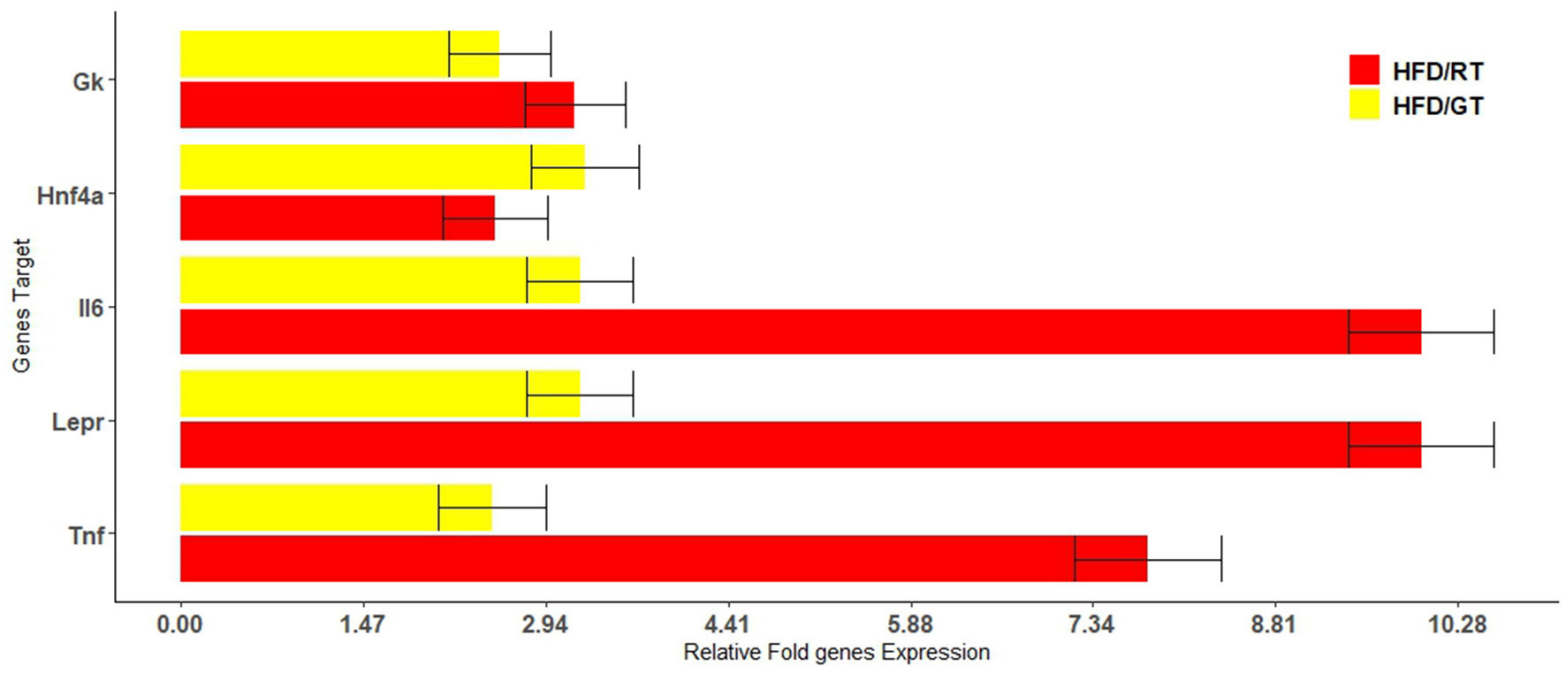
Relative changes in the fold expression of the genes involved in rat fatty liver pathways with a p-value cutoff of 0.05. The analysis was performed in genes differentially expressed with respect to the HFD control group in dependence on the nutrition supplementation with red tomatoes (red bars) and golden tomatoes (yellow bars).

The microarray analysis of 84 target genes (Supplementary Figure 1) involved in rat fatty liver was performed in biological triplicates for each group as described in section 2.2. The analysis of gene expression in HFD/RT (red bars) and HFD/GT (yellow bars) liver samples were referred to the HFD group. Figure 6 shows gene expression with statistical significance (p-value < 0.05; Supplementary Table 2).

The alimentary supplementation with RT or GT was able to up-regulate, with statistical significance, genes involved in metabolic pathways, adipokine signaling, and inflammatory response. We found that, after administration of GT and RT respectively, GK was up-regulated 2.56 and 3.17 times; Hnf4α resulted up-regulated 3.25 and 2.53 times; Lepr was up-regulated 3.21 and 9.99 times; IL6 was up-regulated 3.21 and 9.99 times; and TNF was up-regulated 2.5 and 7.78 times.

## 4. Discussion

The present study aimed to evaluate the impact of dietary supplementation of red tomato (RT) and golden tomato (GT) in a rat model of NAFLD. We investigated the amelioration of metabolic profile and expression of genes in the liver which plays a key role in adipokine signaling inflammatory response and metabolic pathways to improve hepatic steatosis. The GT represents the harvested version of tomatoes, selected as reported in section 4.1, and its biological activity is yet to be fully discovered. It is characterized by different compositions, in terms of phytochemicals, with higher content of naringenin and chlorogenic acid and lower content of carotenoids and lycopene with respect to RT.

The dietary administration of RT was expected to show metabolic and hepatoprotective effects as they are rich in antioxidants, mainly carotenoids and lycopene (24).

Carotenoids are made up of conjugated double bonds in their structure; they have a high reducing capability by transferring electrons, which gives them antioxidant properties responsible for reducing the risk of atherosclerosis, cancers, and NAFLD (25). The liver is a major site for the storage of β-carotenes which have a positive impact on liver pathology. They are recognized for ameliorating hepatic steatosis and liver injury and decreasing liver enzymes (alanine/aspartate aminotransferase) and bilirubin levels. In addition, they are reported to have the ability to improve insulin sensitivity and thus act as a lipid-lowering agent and lipid-soluble antioxidant (26, 27).

In our experiments, RT dietary supplementation was able to revert the body weight gain observed after HFD administration, together with liver steatosis reduction. These effects could be mediated by β- carotene content, given its role in decreasing fat accumulation associated with a lower risk of NAFLD development (28, 29). Moreover, β-carotene is described to be able to alleviate dyslipidemia according to our results about the significant reduction of TG and LDL Chol plasma levels observed after RT administration (30). Lycopene is the other main pigment present in red tomatoes. It is well-known for decreasing the levels of serum TG, LDL Chol, and FFAs, and for increasing HDL Chol (31). The effects of lycopene at the hepatic level consist of a reduction of steatosis and increases in the expression of antioxidant enzymes (15, 25). These effects have been evidenced in HFD/RT rats with respect to the HFD group, suggesting that the beneficial effects of the RT dietary supplementation are mediated by β-carotenes and lycopene contents.

The novelty of our approach was to evaluate the effects of dietary administration of GT, which is often thrown after harvesting, as a functional food. The intake of GT, rich in naringenin and ChA, was expected to improve metabolic profile and liver steatosis.

Among flavonoids, naringenin has attracted the interest of medicinal biologists and chemists exhibiting a broad range of biological and pharmacological activities such as antioxidant, anti-allergic, antibacterial, anti-inflammatory, antimutagenic, and antiproliferative effects in different cancer cell lines (32, 33). It is reported that GT has lipid-lowering and insulin-like properties. In addition, its protective effects in metabolic and cardiovascular contexts have demonstrated its ability to ameliorate the metabolic syndrome features in terms of antiadipogenic effects due to the increase of fatty acid oxidation and decrease of *de novo* lipogenesis (34, 35). These reports are in line with our results that show the decrease in biometrics and the reduction of steatosis with the loosing of macro vesicular phenotype after GT dietary supplementation. In the same way, the reduction of TG and LDL Chol and the significant increase of HDL Chol with respect to the HFD diet supported the hypothesis of naringenin-mediated beneficial effect (36).

Chlorogenic acid is one of the most available polyphenol compounds in food and is considered a well-known antioxidant agent (37). It can be found ubiquitously in plants, such as apples, coffee, herbal tea, and RT, but it is more abundant in GT (81%) (38).

Accumulating evidence has demonstrated that ChA exhibits many biological properties, including antibacterial, antioxidant, and anticarcinogenic activities. Furthermore, its metabolic effects have been described; it plays a crucial role in glucose and lipid metabolism, acting as a hypoglycemic and hypolipidemic agent, as reported in models of rats fed a high-cholesterol diet (39). Chlorogenic acid exerts beneficial effects, especially on obesity-related liver steatosis and insulin resistance. It can attenuate both the reduced plasma concentrations of HDL and increased total cholesterol and plasma concentrations of LDL (40).

Our experiments showed that the effects of supplementation with GT in HFD rats are effective in ameliorating the lipidic profile (reduction of TG and LDL Chol and increase of HDL Chol), together with glucose homeostasis (reduction of AUC and fasting glucose) and the insulin resistance. Moreover, the observed reduction of body weight in the HFD/GT group could be ascribable in part to the ChA content in GT, given that this phytonutrient exerts a beneficial influence on obesity-related liver steatosis, blocking diet-induced weight gain (41).

To understand the molecular mechanisms related to the observed modifications of metabolic profile and liver histological characteristics, we evaluated the tissue expression of genes involved in metabolic pathways, adipokine signaling, and inflammatory response. As expected, several genes were differentially expressed in HFD/GT and HFD/RT with respect to HFD. However, we focused our attention on genes markedly upregulated in both treatments with GT and RT.

We found higher levels of *Il6* and *Tnf* transcripts, both of which are involved in the inflammatory response in GT and RT groups, than HFD. However, in the GT group, their levels were lower (respectively 3.2 times and 2.5 times) than in the RT group (respectively 9.9 times and 7.7 times). These results could be explained as an outcome of rapid mobilization of TG from the liver during the GT and RT treatment, observed in terms of lipid liver content, which could be linked to the increase in inflammation. However, it is expected that the overall anti-inflammatory effect of RT/GT supplementation is beneficial due to the lycopene, β-carotene (42), and mainly naringenin (43) and ChA (16) content of these matrices.

Interestingly, we found higher levels of *Hnf4*α in HFD/GT (3.25 times) and in HFD/RT (2.53 times) compared to the HFD group. *Hnf4*α is a nuclear receptor that plays a crucial role in hepatic lipid homeostasis, regulating the transcription of genes involved in the secretion of VLDL, such as apolipoprotein B (44). Hepatic *Hnf4*α expression is markedly reduced in diabetes, obesity, and NASH. It has been reported that after HFD feeding, there is a reduction in mRNA and protein levels in hepatic *Hnf4*α and its cytoplasmatic retention (45). Treatments that can induce *Hnf4*α expression or activation were investigated, leading to the discovery of strong *Hnf4*α agonists that can control fat deposition in the liver. Since hepatic *Hnf4*α is repressed in NASH, the treatment with GT and RT can increase its liver expression, which could be useful in the prevention and progression of NAFLD (46).

Furthermore, the gene expression analysis showed increased levels of Leptin receptor (*Lepr)* in HFD/GT (3.21 times) and in HFD/RT (9.99 times of *p*-value 0.005) compared to the HFD group. *Lepr* has been reported as a possible target gene being upregulated by metformin which may enhance leptin sensitivity in the liver to alleviate steatosis. Hepatic leptin resistance is a key determinant of lipid accumulation in the liver (47). Much evidence indicates that LEPR serves as a novel biomarker for leptin sensitivity, and the augmentation of LEPR is related to the reduction of steatosis (48). However, increased *Lepr* levels have been positively correlated with HDL Chol and serum adiponectin levels (47). In line with the literature, several effects observed after GT and RT supplementation could be reconducted to *Lepr* up-regulation. Interestingly, enhancing leptin response improves liver function (by reducing fat deposits) and improves glycerol import into hepatocytes to maintain glucose and lipid metabolism (18). Our experimental data showed that in rats fed with RT and GT, the expression of the enzyme glycerol kinase (GK) increased. Glycerol kinase is a crucial enzyme in the interface of fat and carbohydrate metabolism by converting glycerol to glycerol 3-phosphate (G3P) in a reaction dependent on ATP availability (19). In rat models of hypercholesterolemia, rats receiving chickpeas re-established the liver glycogen deposition compared to the control group; this effect has been correlated to the increase in GK activity (49). Our study reported the reduction of body weight in rats fed with GT and RT compared to the HFD group. In addition, we found a decrease in TG levels in HFD/GT and HFD/RT groups compared to rats fed with HFD alone. The microarray analysis reported higher levels of *Gk* in HFD/GT (2.56 times) and HFD/RT (3.17 times) than in the group fed with HFD alone. The high expression of *Gk* could correlate to the reduction of TG that was used for gluconeogenesis. In this context, the rats receiving GT or RT solution could re-establish the liver glycogen deposition through *Gk* activity.

In conclusion, our results showed that the dietetic administration of red and golden tomatoes in the NAFLD rat model was beneficial in terms of body weight loss, reversion of lipid liver accumulation, and lipidic and glycemic profiles. However, the beneficial effects were more evident in the group that received GT, suggesting the possibility of introducing GT, together with RT, in the diet. The liver expression of genes such as *Hnf4, Lepr*, and *Gk*, which are key modulators of liver lipid metabolism, was observed to depend on the dietary intake of red or golden tomatoes. It was reported that naringenin exerts antifibrotic effects, inhibiting the trans-differentiation of hepatic stellate cells and reducing collagen synthesis (50). In line with this evidence, the GT diet could play a role in the prevention of NAFLD development and also antagonize the progression to NASH, prodromic to liver fibrosis. Further studies are necessary to evaluate the effects of the co-administration of red and golden tomatoes, but our results suggest that this type of co-supplementation could represent a dietary strategy to prevent NAFLD development and progression.

## Data availability statement

The datasets presented in this study can be found in online repositories. The names of the repository/repositories and accession number(s) can be found below:

- Glicerol Kinase (GK) [*Rattus norvegicus* (Norway rat)] NM 024381.2.
  Gene ID: 79223.
  https://www.ncbi.nlm.nih.gov/nuccore/NM_024381.2.
- Hepatocyte Nuclear Factor 4, alpha (HNFa) [*Rattus norvegicus* (Norway rat)] NM 001270931.1.
  Gene ID: 25735.
  https://www.ncbi.nlm.nih.gov/nuccore/NM_001270931.1.
- Interleukin 6 (Il6) [*Rattus norvegicus* (Norway rat)] NM 012589.2.
  Gene ID: 24498.
  https://www.ncbi.nlm.nih.gov/nuccore/NM_012589.2.
- Lepr leptin receptor [*Rattus norvegicus* (Norway rat)] NM 012596.2.
  Gene ID: 24536.
  https://www.ncbi.nlm.nih.gov/nuccore/NM_012596.2.
- Tumor Necrosis Factor (Tnf) [*Rattus norvegicus* (Norway rat)] NM 012675.3.
  Gene ID: 24835
  https://www.ncbi.nlm.nih.gov/nuccore/NM_012675.3.

## Author contributions

RMP, DC, DD, and SG contributed to conceptualization. RMP, RZ, GGa, GD, AJ, GL, GGi, PS, FR, and SG contributed to data curation. GGa and GD contributed to software and statistical analysis. GD, DD, and SG contributed to funding acquisition. RMP, RZ, GGa, AJ, GL, GGi, GF, FR, DC, DD, and SG contributed to the investigation. RMP, RZ, GGa, GL AJ, FR, DD, and SG contribute to methodology. GF and SG supervised the drafting of the manuscript. RMP and RZ contributed to validation. RMP, DC, RZ, DD, and SG wrote the original draft. RMP and SG reviewed and edited the draft. All authors contributed to the article and approved the submitted version.
