# Supplementary material for: Red and golden tomato administration improves fat diet-induced hepatic steatosis in rats by modulating HNF4α, Lepr, and GK expression
Source: Front Nutr. 2023 Sep 1;10:1221013. doi: 10.3389/fnut.2023.1221013 (PMC10505813; doi:10.3389/fnut.2023.1221013)
Supplement: Supplementary file 1 [file Table_1.docx]

**Table S1. List of 84 target genes of RT2 Profiler PCR Array (96-Well Format) Rat Fatty Liver (Car. No. 330231 PARN-157ZA)**

|  |  | |  | | | |  |
| --- | --- | --- | --- | --- | --- | --- | --- |
|  | |  | |  |  |  |  |
| 1. ABCA1 | | NM_178095 | | 32. G6PC | NM_013098 | 61. PIK3CA | NM_133399 |
| 1. ABCG1 2. ACACA | | NM_053502  NM_022193 | | 33. G6PD | NM_017006 | 62. PIK3R1 | NM_013005 |
| 1. ACADL | | NM_012819 | | 34. GCK | NM_012565 | 63. PKLR | NM_012624 |
| 1. ACLY | | NM_016987 | | 35. GK | NM_024381 | 64. PPA1 | NM_001100834 |
| 1. ACOX1 | | NM_017340 | | 36. GSK3B | NM_032080 | 65. PPARA | NM_013196 |
| 1. ACSL5 | | NM_053607 | | 37. HMGCR | NM_013134 | 66. PPARD | NM_013141 |
| 1. ACSM3 | | NM_033231 | | 38. HNF4A | NM_022180 | 67. PPARG | NM_013124 |
| 1. ADIPOR1 | | NM_207587 | | 39. IFNG | NM_138880 | 68. PPARGC1A | NM_031347 |
| 1. ADIPOR2 | | NM_001037979 | | 40. IGF1 | NM_178866 | 69. PRKAA1 | NM_019142 |
| 1. AKT1 | | NM_033230 | | 41. IGFBP1 | NM_013144 | 70. PTPN1 | NM_012637 |
| 1. APOA1 | | NM_012738 | | 42. IL10 | NM_012854 | 71. RBP4 | NM_013162 |
| 1. APOB | | NM_019287 | | 43. IL1B | NM_031512 | 72. RXRA | NM_012805 |
| 1. APOC3 | | NM_012501 | | 44. IL6 | NM_012589 | 73. SCD | NM_139192 |
| 1. APOE | | NM_138828 | | 45. INSR | NM_017071 | 74. SERPINE1 | NM_012620 |
| 1. ATP5C1 | | NM_053825 | | 46. IRS1 | NM_012969 | 75. SLC27A5 | NM_024143 |
| 1. CASP3 | | NM_012922 | | 47. LDLR | NM_175762 | 76. SLC2A1 | NM_138827 |
| 1. CD36 | | NM_031561 | | 48. LEPR | NM_012596 | 77. SLC2A2 | NM_012879 |
| 1. CEBPB | | NM_024125 | | 49. LPL | NM_012598 | 78. SLC2A4 | NM_012751 |
| 1. CNBP | | NM_022598 | | 50. MAPK1 | NM_053842 | 79. SOCS3 | NM_053565 |
| 1. CPT1A | | NM_031559 | | 51. MAPK8 | NM_053829 | 80. SREBF1 | NM_001276707 |
| 1. CPT2 | | NM_012930 | | 52. MLXIPL | NM_133552 | 81. SREBF2 | NM_001033694 |
| 1. CYP2E1 | | NM_031543 | | 53. MTOR | NM_019906 | 82. STAT3 | NM_012747 |
| 1. CYP7A1 | | NM_012942 | | 54. NDUFB6 | NM_001106646 | 83. TNF | NM_012675 |
| 1. DGAT2 | | NM_001012345 | | 55. NFKB1 | NM_001276711 | 84. XBP1 | NM_001004210 |
| 1. FABP1 | | NM_012556 | | 56. NR1H2 | NM_031626 |  |  |
| 1. FABP3 | | NM_024162 | | 57. NR1H3 | NM_031627 |  |  |
| 1. FABP5 | | NM_145878 | | 58. NR1H4 | NM_021745 |  |  |
| 1. FAS | | NM_139194 | | 59. PCK2 | NM_001108377 |  |  |
| 1. FASN | | NM_017332 | | 60. PDK4 | NM_053551 |  |  |
| 1. FOXA2 | | NM_012743 | |  |  |  |  |
|  | |  | |  |  |  |  |
