# Supplementary material for: Red and golden tomato administration improves fat diet-induced hepatic steatosis in rats by modulating HNF4α, Lepr, and GK expression
Source: Front Nutr. 2023 Sep 1;10:1221013. doi: 10.3389/fnut.2023.1221013 (PMC10505813; doi:10.3389/fnut.2023.1221013)
Supplement: Supplementary file 2 [file Table_2.docx]

**Supplementary Table 2.** Relative changes in the fold expression of the genes differentially expressed with respect to HFD control group in dependence of the nutrition supplementation with golden tomatoes (HFD/GT)), and red tomatoes (HFD/RT) and p-value genes.

| Gene | Fold change in HFD/GT group | p-value  HFD/GT group | Fold change in HFD/RT group | p-value  HFD/RT group |
| --- | --- | --- | --- | --- |
| GK | 2,56 times | 0,03 | 3,17 times | 0,017 |
| HNF4α | 3,25 times | 0,018 | 2,53 times | 0,022 |
| IL6 | 3,21 times | 0,016 | 9,99 times | 0,005 |
| Tnf | 2,5 times | 0,03 | 7,78 times | 0,008 |
| Lepr | 3,21 times | 0,016 | 9,99 times | 0,005 |
