# Supplementary figures and images for: Red and golden tomato administration improves fat diet-induced hepatic steatosis in rats by modulating HNF4α, Lepr, and GK expression
Source: Front Nutr. 2023 Sep 1;10:1221013. doi: 10.3389/fnut.2023.1221013 (PMC10505813; doi:10.3389/fnut.2023.1221013)

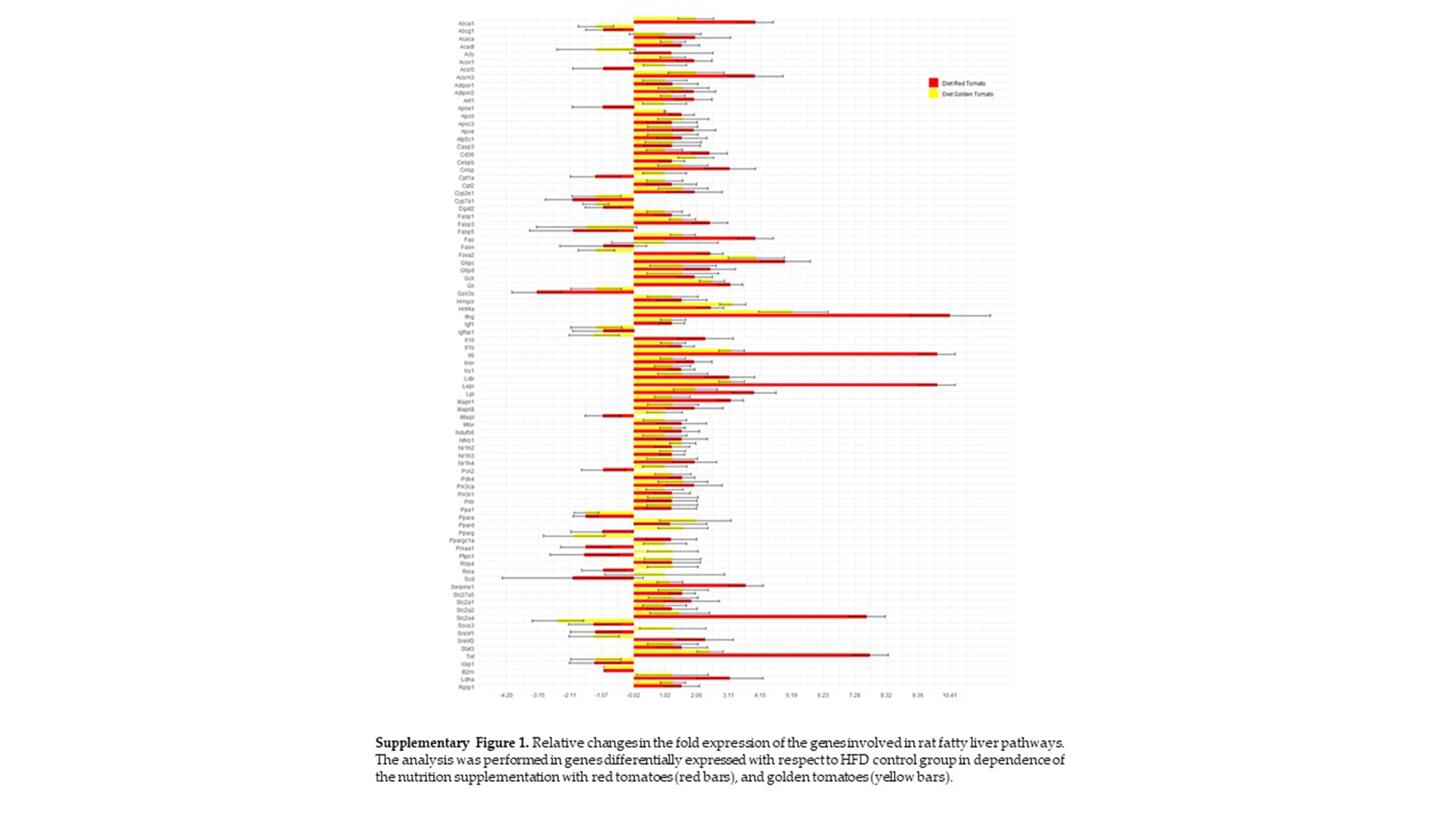

Supplement: Supplementary file 3 [file Image_1.JPEG]
